# Supplementary material for: Many will enter, few will win: Cost and sensitivity of exploratory dynamics
Source: Biophys J. Author manuscript; Available in PMC 2026 Mar 5. (PMC12958470; doi:10.1016/j.bpj.2025.09.007)
Supplement: 1 [file NIHMS2142448-supplement-1.pdf]

SUPPLEMENTARY MATERIAL
Many Will Enter, Few Will Win: Cost and Sensitivity of Exploratory Dynamics

Elena F. Koslover,* Milo M. Lin, and Rob Phillips

Appendix A. COARSE-GRAINING REALISTIC PROOFREADING SCHEMES

The probabilistic approach described here can be generalized to more complex irreversible proofreading systems by mapping to the general scheme shown in Fig. S1A. In particular, this includes translational proofreading systems with additional transitions both before and after the energy-consuming step. Such schemes (see Fig. S1B as an example) have been used to summarize experimental data on the kinetics of translational elongation in past work [1–4]. The generalized scheme considered here uses an analogous coarse-graining procedure to that described in recently published work by Igoshin, *et al* [5], which trims states and non-energy-consuming loops using splitting probabilities and mean-first-passage times between remaining milestone states.

In Fig. S1A, the one-way arrows denote reactions where the reverse rates are negligible in their effect on splitting probabilities. The red arrows mark the only energy-consuming steps (eg: GTP hydrolysis), and the energy-consumption and release arrows denote transitions along distinct pathways. Release from the high-energy (purple) macrostate is assumed to return the system to the same (green) microstate, regardless of which tRNA is released.

This system can be treated as a heterogeneous continuous-time random walk [6] with Markovian (albeit not constant-rate) transitions on a simplified coarse-grained network of states. We can define the splitting probabilities p_c, p_w for transitioning out of the R into the RC* or RW* states respectively. Specifically, p_c is the probability for a particle starting in the initial (green) state to first reach the compound state RC* before it ever reaches state RW*. We can also define the probability p_{pc} that a particle in the RC* macrostate will first transition to the elongated state, before a release occurs, and the probability $p_{uc} = 1 - p_{pc}$ for the opposite case.

With these definitions, we can proceed using the same analysis as before. Consider each individual 'interaction' event with no intermediate returns to the initial state. The probability that an interaction passes through the RC* state and results in elongation is $p_c p_{pc}$. The probability that it passes through the RC* state but results in release is $p_c p_{uc}$.

The error rate f and the number of excess transitions over the energy-consuming pathway $\langle n \rangle$, can then be written analogously to Eq. 6, 8:

$$f = \frac{p_w p_{pw}}{p_c p_{pc}}, \tag{S1a}$$

$$\langle n \rangle = \frac{p_c p_{uc} + p_w p_{uw}}{p_c p_{pc} + p_w p_{pw}} \tag{S1b}$$

We note that the scheme in Fig. S1A is equivalent to a substrate-selective Michaelis-Menten enzymatic reaction. For such reactions, the accuracy has previously been expressed as a ratio of the catalytic efficiencies $k_{\text{cat}}/K_{\text{m}}$ for the cognate versus noncognate substrates [7, 8]. The error rate in Eq. S1a is directly equivalent to such an expression.

*Author for correspondence: ekoslover@ucsd.edu

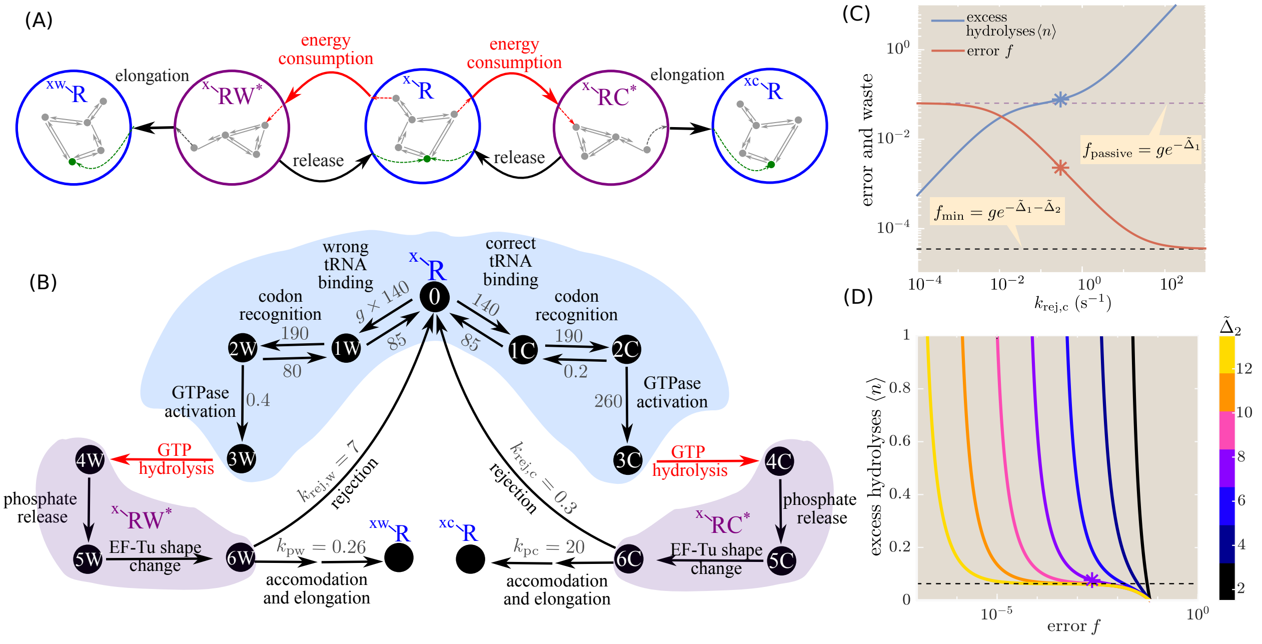

FIG. S1: **Alternate schemes for translational proofreading with a single energy-consuming step.** (A) General schematic, where each circle corresponds to a compound state containing no internal driven transitions. Red arrows represent the only energy-consuming steps in the system. (B) Example of more complex proofreading scheme, employed in Ref. [1, 3, 4] to describe experimental measurements of ribosomal translation. Shaded regions delineate coarse-graining to the compound states in (A). Rate constants (gray) from Ref. [3]. All rate constant units are in $\sec^{-1}$, except for the binding rate in $\mu M^{-1}s^{-1}$. (C) Plot of error f and energetic cost in terms of excess hydrolyses $\langle n \rangle$, for the realistic ribosomal translation model and parameters given in (B), with varying rejection rates. The rejection rate for the wrong pathway is set to $k_{\text{rej,w}} = k_{\text{rej,c}} e^{\tilde{\Delta}_2}$, with the discrimination factor $\tilde{\Delta}_2 = 7.5$, and all other rates are kept constant throughout. Dashed lines indicate limits for error rate with slow and rapid rejection. (D). Plot of excess hydrolysis cost $\langle n \rangle$ versus the error rate f, for different values of the proofreading discrimination factor $\tilde{\Delta}_2$, using the realistic model and parameters from (B). Dashed line marks the plateau region $\langle n \rangle \approx g e^{-\tilde{\Delta}_1}$. Stars in (C) and (D) mark the specific measured parameters from Ref. [3].

A.1. Realistic model and parameters for ribosomal translation

For the specific scheme illustrated in Fig. S1B, employed in Ref. [1, 3], the "R" macrostate can be considered to include the initial binding and codon recognition transitions, as well as GTPase activation, with the energy-consuming exit from this macrostate corresponding to GTP hydrolysis. Since the GTPase activation is assumed to be effectively irreversible, the splitting probability out of the initial compound state is simply the probability that hydrolysis with the correct tRNA occurs before hydrolysis with the wrong tRNA (state 3C is reached before state 3W). This probability can be computed through coarse-graining of the reaction scheme as follows.

First, we find the probability $\hat{p}_{02}$ that a system starting at state 0 hits the 2C state before the 2W state. This can be done by considering each time the system leaves state 0 as the start of an independent path. Each such path must either reach state $2C$ (with probabilistic weight $p_{01}^{(c)} p_{02}^{(c)}$) or state $2W$ (with probabilistic weight $p_{01}^{(w)} p_{02}^{(w)}$) or else return to the beginning at state 0. The

resulting probability of hitting $2C$ first is then:

$$\hat{p}_{02}^{(c)} = \frac{p_{01}^{(c)} p_{12}^{(c)}}{p_{01}^{(c)} p_{12}^{(c)} + p_{01}^{(w)} p_{12}^{(w)}}, \tag{S2}$$

where $p_{ij}^{(c)}$ is the splitting probability from state i to adjacent state j with the correct tRNA. Similarly, we find the probability $\hat{p}_{23}$ that a system starting at state 2 hits the 3C state before returning to the 0 state. Again we consider the weight of each path leaving state 2 without returning to it, to get:

$$\hat{p}_{23}^{(c)} = \frac{p_{23}^{(c)}}{p_{23}^{(c)} + p_{21}^{(c)} p_{10}^{(c)}}. \tag{S3}$$

Analogous probabilities are defined for the wrong tRNA. The desired splitting probability for leaving the macrostate entirely (through GTPase activation and hydrolysis) is then

$$p_c = \frac{\hat{p}_{02}^{(c)} \hat{p}_{23}^{(c)}}{\hat{p}_{02}^{(c)} \hat{p}_{23}^{(c)} + \hat{p}_{02}^{(w)} \hat{p}_{23}^{(w)}}. \tag{S4}$$

Note that this approach can be generalized to recursively compute splitting probabilities for any number of interchanging states arranged in a linear array.

To obtain realistic parameters for ribosomal translation, we use the values reported in Ref. [3] for a specific pair of cognate and near-cognate codons (UUU and CUC). The measured values are given in Fig. S1B. Individual rates that were not measured (such as the EF-Tu shape change) follow a prior irreversible transition and do not affect the splitting probabilities of the system. In addition to the rates for a specific 'wrong' tRNA, we need an estimate for the factor g, defining the concentration excess of wrong versus right substrates. A variety of different error rates have been measured for various near-cognate codon and anti-codon pairings [9, 10], while non-near-cognate interactions are almost never observed [1, 11]. We estimate the relevant concentration factor by noting that tRNA carrying the amino acid Lys has been shown to interact with 16 cognate and near-cognate codons, with varying yet measurable frequencies [9]. If each tRNA has some cross-reaction with 1/4 of the possible codons, this would imply that each codon position has a measurable chance of incorporating ~ 5 of the twenty possible amino acids into the chain, through cognate and near-cognate tRNA interactions. Of course, this estimate neglects the varying frequencies of distinct tRNAs *in vivo*, and the variety of translational kinetic rates for each. However, we take a rough average estimate of $g = 4$ for the excess concentration of near-cognate tRNAs bearing the wrong amino acid that can interact with the ribosome sufficiently to proceed through the hydrolysis step and thereby contribute to the error and energetic cost.

Using the numbers in Fig. S1B [3], together with $g = 4$, gives the estimated splitting probabilities $p_c = 0.94, p_w = 0.06$ for passing the hydrolysis step with the right or wrong amino acid. The probabilities of elongation rather than release after hydrolysis are estimated as $p_{pc} = 0.98, p_{pw} = 0.04$, giving an overall error rate of $f \approx 2 \times 10^{-3}$ and a low cost in futile hydrolysis cycles of $\langle n \rangle \approx 0.08$. We note that these numbers imply that $96^{\%}$ of wrong tRNAs are released after hydrolysis, while only $2^{\%}$ of correct tRNAs are released. Thus, this system is in a regime where much of the discrimination occurs on the initial binding step and most of the correct tRNAs that make it through hydrolysis go on to proceed to elongation, with very few futile cycles.

As shown in Fig. S1C,D, this more realistic model of ribosome kinetics engenders a trade-off between energetic cost and accuracy that is analogous to the simple model described in the main

text. When the post-hydrolysis rejection rate is low, the error rate is determined entirely by the initial steps leading to hydrolysis. We can define a discrimination factor for this passive part of the process as:

$$e^{\tilde{\Delta}_1} = \log\left[p_{12}^c \hat{p}_{23}^c / \left(p_{12}^w \hat{p}_{23}^w\right)\right]. \tag{S5}$$

For the simple model in Fig. 1, the discrimination factor $\tilde{\Delta}_1$ approaches the binding energy difference Δ_1 when hydrolysis is slow. For the realistic model in Fig. S1B, where hydrolysis is relatively fast compared to unbinding, the parameter $\tilde{\Delta}_1 \approx 4.1$ serves an analogous role in setting the error rate in the absence of active proofreading ($f \to ge^{-\tilde{\Delta}_1}$).

For the discrimination factor associated with the proofreading step in this generalized model, we define

$$e^{\tilde{\Delta}_2} = \log\left[\left(k_{\text{rej,w}}/k_{\text{pw}}\right) / \left(k_{\text{rej,c}}/k_{\text{pc}}\right)\right]. \tag{S6}$$

This factor incorporates both the difference in release rate (as in the original simple model) and the difference in elongation rate for the correct versus the wrong tRNA. For the measured parameters, it can be estimated as $\tilde{\Delta}_2 \approx 7.5$. In the limit where rejection rates are much higher than elongation rates , the overall error approaches $f \to ge^{-\tilde{\Delta}_1 - \tilde{\Delta}_2}$.

The cost in terms of excess hydrolysis events is related to the error rate in much the same way as for the simple model. As plotted in Fig. S1D, approaching the minimal possible error rate drives up the cost. There is a plateau for intermediate error rates, corresponding to the regime where most wrong tRNAs are rejected while most correct ones move on to elongation. The plateau cost is equal to the passive error rate ($ge^{-\tilde{\Delta}_1}$), and the plateau is wider for larger values of the proofreading discrimination factor $\tilde{\Delta}_2$. For the estimated rate constants used here, the system sits within a plateau region, with moderate error rates and relatively little waste in terms of futile hydrolysis events. This is consistent with past analyses, which indicated that ribosomal translation is optimized for speed and low energy cost rather than maximal accuracy [12].

Given the analogous behavior of this more realistic model and the classic scheme in Fig. 1, we use the parameters $g = 4, \Delta_1 = 4, \Delta_2 = 8$ as the most relevant default values for analyzing the highly simplified model in the main text.

Appendix B. FIDELITY AND ENERGETIC COST FOR REVERSIBLE MODEL

For the model with reversible transitions (Fig. 5) we can define the splitting probabilities for correct and wrong tRNAs in terms of either the rate constants or the transition state energies, as

follows:

$$p_{bc} = \frac{k_b}{(k_b + k_{ur})(1+g)} = \frac{e^{-E_b^\ddagger}}{(e^{-E_b^\ddagger} + e^{-E_2^\ddagger})(1+g)}, \quad p_{bw} = \frac{gk_b}{(k_b + k_{ur})(1+g)} = \frac{ge^{-E_b^\ddagger}}{(e^{-E_b^\ddagger} + e^{-E_2^\ddagger})(1+g)}, \tag{S7a}$$

$$p_{urc} = \frac{k_{ur}}{(k_b + k_{ur})(1+g)} = \frac{e^{-E_2^\ddagger}}{(e^{-E_b^\ddagger} + e^{-E_2^\ddagger})(1+g)}, \quad p_{urw} = \frac{gk_{ur}}{(k_b + k_{ur})(1+g)} = \frac{ge^{-E_2^\ddagger}}{(e^{-E_b^\ddagger} + e^{-E_2^\ddagger})(1+g)}, \tag{S7b}$$

$$p_{hc} = \frac{k_h}{k_h + k_{u1}} = \frac{e^{-E_h^\ddagger + \epsilon_{\text{drive}}}}{\left(e^{-E_h^\ddagger + \epsilon_{\text{drive}}} + e^{-E_b^\ddagger}\right)}, \quad p_{hw} = \frac{k_h}{k_h + k_{u1w}} = \frac{e^{-E_h^\ddagger + \epsilon_{\text{drive}}}}{\left(e^{-E_h^\ddagger + \epsilon_{\text{drive}}} + e^{-E_b^\ddagger + \Delta_1}\right)}, \tag{S7c}$$

$$p_{rc} = \frac{k_r}{k_r + k_{u2} + k_p} = \frac{e^{-E_h^\ddagger}}{\left(e^{-E_h^\ddagger} + e^{-E_2^\ddagger} + e^{-E_p^\ddagger}\right)}, \quad p_{rw} = \frac{k_{rw}}{k_{rw} + k_{u2w} + k_p} = \frac{e^{-E_h^\ddagger - \Delta_1}}{\left(e^{-E_h^\ddagger - \Delta_1} + e^{-E_2^\ddagger} + e^{-E_p^\ddagger - \Delta_2}\right)}, \tag{S7d}$$

$$p_{pc} = \frac{k_p}{k_r + k_{u2} + k_p} = \frac{e^{-E_p^\ddagger}}{\left(e^{-E_h^\ddagger} + e^{-E_2^\ddagger} + e^{-E_p^\ddagger}\right)}, \quad p_{pw} = \frac{k_p}{k_{rw} + k_{u2w} + k_p} = \frac{e^{-E_p^\ddagger - \Delta_2}}{\left(e^{-E_h^\ddagger - \Delta_1} + e^{-E_2^\ddagger} + e^{-E_p^\ddagger - \Delta_2}\right)}, \tag{S7e}$$

where $k_h = k_h^0 + \alpha = k_h^0 e^{\epsilon_{\text{drive}}}$ is the total hydrolysis rate.

We consider individual interactions of a tRNA with a ribosome, each of which involves leaving the empty R state of the ribosome and eventually returning to it (possibly with a longer peptide chain), without any intermediate visits to that state. Each interaction can be resolved through either unbinding, release from the high-energy intermediate state, or elongation.

For a system that reaches the high-energy RC^* state, the probabilistic weight of all paths with exactly i hydrolysis transitions since the start of the interaction is:

$$w_{i,c} = p_{urc}(p_{rc}p_{hc})^i + p_{bc}p_{hc}(p_{rc}p_{hc})^{i-1}, \tag{S8}$$

with an analogous expression $w_{i,w}$ for a system in the RW^* state. The probability that the interaction resolves in elongation with the correct or the wrong tRNA ($p_{el,c}, p_{el,w}$) is then

$$\begin{aligned} p_{\text{el,c}} &= p_{urc}p_{pc} + \sum_{i=1}^{\infty} w_{ic}p_{pc} = \frac{(p_{urc} + p_{bc}p_{hc})p_{pc}}{1 - p_{rc}p_{hc}} \\ p_{\text{el,w}} &= \frac{(p_{urw} + p_{bw}p_{hw})p_{pw}}{1 - p_{rw}p_{hw}}. \end{aligned} \tag{S9}$$

The error rate is given by $f = p_{\text{el,w}}/p_{\text{el,c}}$, yielding Eq. 11.

The average number of hydrolysis steps for an interaction involving the correct tRNA (N_c) is found by summing over the corresponding $w_{i,c}$, multiplied by the probability that the high-energy state resolves with no further hydrolysis transitions: $p_{fc} = 1 - p_{rc}p_{hc}$. The result for both correct and wrong interactions is:

$$\begin{aligned} N_c &= \sum_{i=0}^{\infty} i w_{i,c} p_{fc} = \frac{(p_{bc} + p_{urc}p_{rc})p_{hc}}{1 - p_{rc}p_{hc}} \\ N_w &= \frac{(p_{bw} + p_{urw}p_{rw})p_{hw}}{1 - p_{rw}p_{hw}} \end{aligned} \tag{S10}$$

Finally, we can find $\langle N \rangle$: the average number of driven activation transitions per interaction event, conditional on that event resolving to elongation. Here we multiply by the fraction of activation transitions that proceed along the driven arrow rather than the basal activation process: $\alpha/(k_h^0 + \alpha) = 1 - e^{-\epsilon_{\rm drive}}$, to yield:

$$\langle N \rangle = \left[\frac{N_c + N_w}{p_{el,c} + p_{el,w}} \right] (1 - e^{-\epsilon_{\rm drive}}), \tag{S11}$$

which gives Eq. 12 in the main text.

Appendix C. CATALYTIC CONTROL FROM EXPLORATORY DYNAMICS WITH RESETTING

Below we describe two distinct approaches to computing the steady-state length distribution of a simple model consisting of reversible motion along a linear set of states, with a constant (catalytically controlled) rate of resetting to the origin. The first approach uses weighted path counting, analogously to the proofreading models in the main text, to compute the length at which resetting occurs. This approach is simple enough to compute manually, but is limited to irreversible resetting transitions. The second approach maps the system to an electric circuit, which can be analyzed in its entirety using well-established matrix methods.

C.1. Microtubule length control by irreversible resetting, via path-counting

Here we demonstrate how the approach of adding up probabilistically weighted paths can be extended to larger reaction systems with multiple intermediate states. The system considered here is a simple model of dynamic instability (Fig. 10A), in the limit where the catastrophe transitions are effectively irreversible. For a microtubule that initially starts at length 1, We seek to compute the probability $P_L^{\rm cat}$ that catastrophe occurs from the L state. For simplicity, we assume that the forward, reverse, and catastrophe rates are constant for all states.

We begin by defining the splitting probabilities at each state. For state $i > 1$, the probabilities of stepping forward and backward are, respectively, $p_f = (k_f + \alpha)/(k_f + \alpha + k_r + k_c), p_r = k_r/(k_f + \alpha + k_r + k_c)$. From each such state there is also a catastrophe probability $p_c = k_c/(k_f + \alpha + k_r + k_c)$. For state $i = 1$, the probability of stepping forward is simply 1. In the case where $p_r \to 0$, the system steps forward until catastrophe is reached. The probability this happens in state i is then the product of forward-stepping probabilities for the first $i-1$ steps times the catastrophe probability: $P_i^{\rm cat} = p_f^{i-2}(1 - p_f)$. This probability is normalized over $i > 1$.

For the case with substantial reversals, the paths can include any number of back and forth steps, so long as they never go below 0 and end in the $i^{\rm th}$ state. These paths can be conveniently enumerated using stone-fence diagrams and continued fractions, as previously employed for computing statistics of semiflexible polymers [13, 14]. Specifically, we define w_i^+ as the total weight of all paths that start at state i and never go below it. This quantity can be expanded as

$$\begin{aligned} w_i^+ &= 1 + p_f w_{i+1}^+ p_r + (p_f w_{i+1}^+ p_r)^2 + \ldots = \frac{1}{1 - p_f p_r w_{i+1}^+}, \quad i > 1, \\ w_1^+ &= \frac{1}{1 - p_r w_{i+1}^+}. \end{aligned} \tag{S12}$$

Here, the first term is a path of length 0, the second includes all paths that step up from the ith level only once (but can meander arbitrarily at $i + 1$ and above), the second term corresponds to

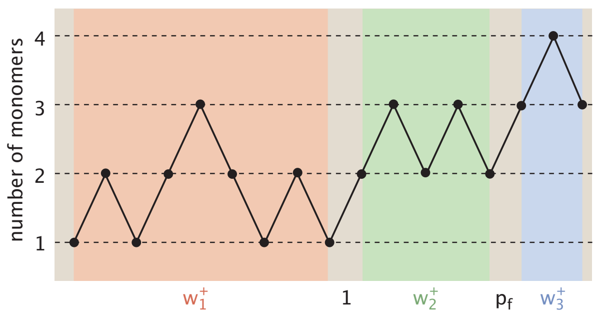

FIG. S2: Stone-fence diagram illustrating example path starting in state 1 and ending in state $i = 4$. Levels correspond to the sequential state i (eg: length of a microtubule in monomers). The path is decomposed into components that end at the last visit to each level, and the probabilistic weights of each component are given beneath.

paths that step up above the ith level twice, and so on. Because we allow the states to go infinitely high, we must have $w_i^+ = w^+$, a constant for all states $i > 1$. We can then get the closed-form expression:

$$w^+ = (1 - \sqrt{1 - 4p_f p_r})/(2p_f p_r). \tag{S13}$$

Any path starting at 1 and ending at state i (with no intervening catastrophes) can be decomposed into the following sequential components: the part of the path up to its last visit at 1, then a step up to 2, the next part of the path up until its last visit at 2, then a step up to 3 and so on until you reach the last section of the path that starts and ends at i and never goes beneath it. This decomposition for an example path is illustrated in Fig. S2. The resulting total weight is then multiplied by p_c to compute the probability that catastrophe happens at state i:

$$\begin{aligned} P_i^{\text{cat}} &= (w_1^+ \cdot 1 \cdot w_2^+ \cdot p_f \cdot w_3^+ \cdot p_f \ldots \cdot w_i^+)p_c \\ &= p_c w_1^+ w^+ (w^+ p_f)^{i-2} = (1 - w^+ p_f)(w^+ p_f)^{i-2}, \end{aligned} \tag{S14}$$

where the last expression accounts for the normalization of the probabilities added up over all states from 2 onwards.

This geometric series skews towards longer lengths when the forward stepping probability p_f becomes high. This probability represents a balance between the forward stepping rate versus reversal or catastrophe. At equilibrium, the catastrophe can only be effectively irreversible if the energies associated with longer-length states are much higher than shorter ones, implying $k_f/k_r \ll 1$. If there is little driving in the system ($\alpha \ll k_r$) then this means $p_f \to 0$ and the distribution of lengths becomes peaked at 1, regardless of the rate of catastrophe. On the other hand, if the system is strongly driven, then $w^+ \to 1$ and we recover the limit with unidirectional stepping, discussed above. The distribution is then determined by $p_f \approx \alpha/(\alpha + k_c)$, with the average length at catastrophe given by $\langle L_{\text{cat}} \rangle \to (2-p_f)/(1-p_f) \to \alpha/k_{\text{cat}}$ for large α. Thus, active driving allows the steady-state microtubule length to be linearly sensitive to the level of catalyst present, ensuring catalytic control.

Finally, we note that because the catastrophe rate is the same from each state, the distribution of length upon catastrophe is directly proportional to the steady-state length distribution: $P_i = \gamma P_i^{\text{cat}}$, where $\gamma = 1 - P_1$ is an appropriate normalization constant.

C.2. Microtubule catalytic control via the circuit mapping

In terms of the nth mesh current shown in Fig. 10B, the voltage equation taken along the path of the nth battery is:

$$P_{n+1}e^{\beta G_{n+1}} - P_n e^{\beta G_n} = \frac{\alpha}{k_f} P_n e^{\beta G_n} - R_n I_n. \tag{S15}$$

where $R_n = e^{\beta G_n}/k_f$ and $G_n = nG$. Note that $e^{\beta G} = k_b/k_f$, where k_f and k_b are the equilibrium forward and backward rates, respectively. Using these definitions, we can solve for the probability of the $(n+1)$th state in terms of the previous state probability and current:

$$P_{n+1} = \Big(1 + \frac{\alpha}{k_f}\Big)e^{-\beta G} P_n - I_n \frac{e^{-\beta G}}{k_f} \tag{S16}$$

Taking the potential difference from state $n+1$ and state 1 along the catastrophe path:

$$P_1 e^{\beta G_1} - P_{n+1}e^{\beta G_n+1} = -R_{\text{cat},n}(I_n - I_{n+1}), \tag{S17}$$

Where $R_{\text{cat},n} = e^{\beta G_{n+1}}/k_{\text{cat}} = R_{n+1}(\frac{k_f}{k_{\text{cat}}})$. Therefore, the $(n+1)$th current is:

$$I_{n+1} = I_n - k_{\text{cat}} P_{n+1} + k_{\text{cat}} P_1 e^{-\beta G_n} \tag{S18}$$

In vector notation, the recursive probability and current equations become:

$$\begin{bmatrix} 1 & 0 \\ k_{\text{cat}} & 1 \end{bmatrix} \begin{bmatrix} P_{n+1} \\ I_{n+1} \end{bmatrix} = \begin{bmatrix} \Big(1 + \frac{\alpha}{k_f}\Big)e^{-\beta G} & -\frac{e^{-\beta G}}{k_f} \\ 0 & 1 \end{bmatrix} \begin{bmatrix} P_n \\ I_n \end{bmatrix} + \begin{bmatrix} 0 \\ \frac{k_{\text{cat}} P_1}{e^{\beta G_n}} \end{bmatrix} \tag{S19}$$

Multiplying both sides by the inverse of the right-hand-side matrix, the recursion relation is:

$$\begin{bmatrix} P_{n+1} \\ I_{n+1} \end{bmatrix} = M \begin{bmatrix} P_n \\ I_n \end{bmatrix} + \begin{bmatrix} 0 \\ \frac{k_{\text{cat}} P_1}{e^{\beta G_n}} \end{bmatrix} \tag{S20}$$

where the transition matrix M is given by:

$$M = \begin{bmatrix} \Big(1 + \frac{\alpha}{k_f}\Big)e^{-\beta G} & -\frac{e^{-\beta G}}{k_f} \\ -k_{\text{cat}}\Big(1 + \frac{\alpha}{k_f}\Big)e^{-\beta G} & k_{\text{cat}}\frac{e^{-\beta G}}{k_f} + 1 \end{bmatrix} \tag{S21}$$

The probability and current of state n in terms of those of state 1 is thus:

$$\begin{bmatrix} P_{n+1} \\ I_{n+1} \end{bmatrix} = M^n \begin{bmatrix} P_1 \\ I_1 \end{bmatrix} + \sum_{k=0}^{n-1} (e^{\beta G} M)^k \begin{bmatrix} 0 \\ k_{\text{cat}} P_1 e^{-\beta G n} \end{bmatrix} \tag{S22}$$

Diagonalizing M:

$$M = V \begin{bmatrix} \lambda_- & 0 \\ 0 & \lambda_+ \end{bmatrix} V^{-1} \tag{S23}$$

Where the columns of V are the eigenvectors of M and λ_- and λ_+ are the eigenvalues of M:

$$\lambda_\pm = \frac{e^{-\beta G}}{2k_f}\left(\alpha + k_f(1+e^{\beta G}) + k_{\text{cat}} \pm \sqrt{(\alpha + k_f - k_f e^{\beta G})^2 + k_{\text{cat}}(2\alpha + 2(1+e^{\beta G})k_f + k_{\text{cat}})}\right) \tag{S24}$$

Which simplifies to the value given in the text:

$$D = -\ln \lambda_- = -\ln [1 - \frac{\sqrt{(\alpha + k_{\text{cat}} + k_f - k_r)^2 + 4k_{\text{cat}}k_r} - (\alpha + k_{\text{cat}} + k_f - kr)}{2k_r}] \tag{S25}$$

Note that $\lambda_- \leq 1$ whereas $\lambda_+ \geq 1$.

The transfer matrix equation is then

$$\begin{bmatrix} P_{n+1} \\ I_{n+1} \end{bmatrix} = V \begin{bmatrix} \lambda_-^n & 0 \\ 0 & \lambda_+^n \end{bmatrix} V^{-1} \begin{bmatrix} P_1 \\ I_1 \end{bmatrix} + \sum_{k=0}^{n-1} e^{\beta kG} V \begin{bmatrix} \lambda_-^k & 0 \\ 0 & \lambda_+^k \end{bmatrix} V^{-1} \begin{bmatrix} 0 \\ k_{\text{cat}} P_1 e^{-\beta Gn} \end{bmatrix} \tag{S26}$$

Expanding this expression and taking the geometric sum yields P_n:

$$P_n = P_1 e^{-\beta G(n-1)} \frac{k_{\text{cat}}}{k_{\text{cat}} - \alpha(e^{\beta G} - 1)} + A_1 \lambda_-^{n-1} + A_2 \lambda_+^{n-1}, \tag{S27}$$

where the A_1 and A_2 are explicit functions of the elementary parameters. For nonzero k_{cat} the probability monotonically decreases for larger n, thus the coefficient A_2 must be zero. Solving this boundary condition for I_1 and substituting into the expression for A_1, we obtain the length distribution (Eq. 14 in the main text):

$$P_n = \frac{k_{\text{cat}}}{k_{\text{cat}} - \alpha(e^{\beta G} - 1)} P_1 e^{-\beta G(n-1)} + \frac{\alpha(e^{\beta G} - 1)}{\alpha(e^{\beta G} - 1) - k_{\text{cat}}} P_1 e^{-D(n-1)}, \tag{S28}$$

where $D = -\ln \lambda_-$.

For microtubule assembly, the net growth rate at physiological α is $\Delta x(k_f + \alpha - k_r) = 10.4$ microns per second, where the change in length per addition of tubulin dimer $\Delta x = 8/13$ nanometers because each tubulin dimer is 8 nanometers long and 13 dimers complete a single turn of the microtubule helix. Microtubule growth and decay rates are assumed to be equal for interphase and mitosis, which are the same to within experimental noise. The catastrophe rate k_{cat} equals 0.01 per second and 0.1 per second in interphase and mitosis, respectively. The mean filament lengths are 23 (11) and 6(3) microns in interphase and mitosis, respectively. All of these parameters and measurements were obtained from Ref. [15], which reported values for Xenopus egg extract with different amounts of Rhodamine tubulin added; for consistency within our model, we used parameters corresponding to 1mg/ml Rhodamine tubulin. k_r was obtained using the values for tubulin bound to the non-hydrolyzable proxy substrate GMPCPP reported in [16].

From the expression for D, we can see that the mean length and the sensitivity of the mean length to k_{cat} is maximal in the limit that $4k_{\text{cat}}k_r/(\alpha + k_{\text{cat}} + k_f - k_r)^2 << 1$(visualized in Fig. 10C). Expanding the expression for D to first order in this ratio, we obtain:

$$D \approx -\ln \left[1 - \frac{k_{\text{cat}}}{|k_{\text{cat}} + \alpha + k_f - k_r|} \right] \tag{S29}$$

In this limit, the mean length retains linear sensitivity to k_{cat} (that is, the linear approximation to the logarithm is valid) if $\alpha > k_r - k_f + \Delta$, where the minimum buffer Δ is set by the value of k_{cat} because $\frac{k_{\text{cat}}}{k_{\text{cat}}+\Delta}$ must be much less than 1. Therefore, as stated in the main text, the transition from weak (logarithmic) to strong (linear) catalytic regulation occurs when $\alpha > k_r - k_f$, with the sharpness being inversely proportional to k_{cat}.

[1] M. V. Rodnina and W. Wintermeyer, Trends Biochem Sci **26**, 124 (2001).
[2] M. Johansson, E. Bouakaz, M. Lovmar, and M. Ehrenberg, Mol Cell **30**, 589 (2008).
[3] I. Wohlgemuth, C. Pohl, J. Mittelstaet, A. L. Konevega, and M. V. Rodnina, Phil Trans Roy Soc B: Biol Sci **366**, 2979 (2011).
[4] H. S. Zaher and R. Green, Mol Cell **39**, 110 (2010).
[5] O. A. Igoshin, A. B. Kolomeisky, and D. E. Makarov, J Phys Chem Lett **16**, 1229 (2025).
[6] D. S. Grebenkov and L. Tupikina, Phys Rev E **97**, 012148 (2018).
[7] M. Johansson, J. Zhang, and M. Ehrenberg, P Natl Acad Sci **109**, 131 (2012).
[8] M. Lovmar and M. Ehrenberg, Biochimie **88**, 951 (2006).
[9] E. B. Kramer and P. J. Farabaugh, Rna **13**, 87 (2007).
[10] T. Daviter, K. B. Gromadski, and M. V. Rodnina, Biochimie **88**, 1001 (2006).
[11] K. Joshi, L. Cao, and P. J. Farabaugh, Yeast **36**, 35 (2019).
[12] K. Banerjee, A. B. Kolomeisky, and O. A. Igoshin, P Natl Acad Sci **114**, 5183 (2017).
[13] H. Yamakawa, J Chem Phys **59**, 3811 (1973).
[14] A. J. Spakowitz and Z.-G. Wang, Phys Rev E **72**, 041802 (2005).
[15] L. D. Belmont, A. A. Hyman, K. E. Sawin, and T. J. Mitchison, Cell **62**, 579 (1990).
[16] H. Bowne-Anderson, M. Zanic, M. Kauer, and J. Howard, Bioessays **35**, 452 (2013).
